# 5-aminolaevulinic acid-based photodynamic therapy inhibits ultraviolet B-induced skin photodamage

**DOI:** 10.7150/ijbs.31583

**Published:** 2019-08-07

**Authors:** Hui Hua, Jia-wei Cheng, Wen-bo Bu, Juan Liu, Wei-wei Ma, Na Ni, Jian Shi, Bing-rong Zhou, Dan Luo

**Affiliations:** 1 Department of Dermatology, The First Affiliated Hospital of Nanjing Medical University, Nanjing, China;; 2 Institute of Dermatology, Chinese Academy of Medical Science and Peking Union Medical College, Nanjing, China.

**Keywords:** photodamage, ultraviolet radiation, photodynamic therapy, DNA damage, CPDs, p53

## Abstract

To evaluate the photoprotective effect of 5-aminolaevulinic acid-based photodynamic therapy (ALA-PDT) on ultraviolet B (UVB)-induced skin photodamage. *In vivo* experiments, the dorsal skin of hairless mice were treated with ALA-PDT or saline-PDT, and then exposed to 180 mJ/m^2^ UVB. Results showed that the number of sunburn cells and apoptotic cells in the epidermis of ALA-PDT-treated groups at 24 h after UVB irradiation were significantly decreased compared with those in the UVB groups. And the removal rate of CPDs was obviously higher in ALA-PDT-treated groups. At 48 h, the number of Ki67 positive nuclei in ALA-PDT-UVB group was significantly fewer than that in UVB group. Further *in vitro* experiments, human keratinocyte cell line (HaCaT) cells of two groups (one treated with ALA-PDT, the other untreated), were exposed to 60 mJ/m^2^ UVB irradiation. We found 0.5 mmol/L of ALA and 3 J/cm^2^ of red light did not affect the vitality of cells, and could reduce UVB induced apoptosis, accelerate the clearance of CPDs, inhibit proliferation and activate p53. Thus, our data demonstrate that ALA-PDT pretreatment can induce a protective DNA damage response that protects skin cells from UVB-induced photodamages.

## Introduction

Recent clinical and epidemiological studies have shown a rising incidence of skin tumors, with ultraviolet (UV) radiation acting as a high-risk environmental factor that also causes many other skin lesions^1^-^3^, such as erythema, edema, skin pigmentation, dysplasia, photoaging and actinic keratosis^4^-^8^. Many processes, mainly DNA damage, are involved in UV-induced skin photodamage. Ultraviolet radiation B (UVB) is a major cause of DNA damage of epidermal cells, as evidenced by over 90% of non-melanoma skin cancers associated with excessive exposure to UVB radiation^9^. UVB may directly damage DNA, generating several types of pro-mutagenic lesions, like cyclobutane pyrimidine dimers (CPDs, a major type) and pyrimidine (6-4) pyrimidinone photoproducts ((6-4)PPs)^10^-^12^. If not eliminated or repaired immediately, these photoproducts may mutate skin cells and even form tumor cells.^13^ A variety of signaling pathways are involved in DNA damage and repair, like P53 pathway^6^, ^14^, ^15^.

As a noninvasive drug-instrument combination technology, 5-aminolaevulinic acid-based photodynamic therapy (ALA-PDT) is combination of photosensitizer, light irradiation and oxygenation. The mechanism of PDT is that excessive amounts of exogenous ALA leads to accumulation of intracellular PpIX, production of cytotoxic reactive oxygen species (ROS) in the presence of light with appropriate wavelength, and the ultimate cell membrane destruction and apoptosis^16^, ^17^. At present, research focus has shifted to the mechanisms of ALA-PDT treating skin photodamage^18^, ^19^, to be specific, the clearance of aged and mutated skin cells^20^. Our research has previously found that ALA-PDT induces apoptosis of photo-aged skin fibroblasts through oxidative damage^21^. In addition, other mechanisms are also engaged. For example, ALA-PDT can weaken the effect of IL-1α on keratinocyte hyperkeratosis by inhibiting the FGFR2 pathway, which is a mechanism in acne treatment^22^.

ALA-PDT has been used to treat skin tumors, photoaging and other photodamage^17^, ^23^, and prevent various photodamaged dermatoses^24^, ^25^. The first animal experiment, conducted in 1997 by Stender, found that ALA-PDT used on a large surface prevented hairless mouse from UV-induced skin cancers^26^. Caty V *et al.* also found that PDT with topical methyl aminolevulinate (MAL) used on a large surface could prevent BCCs of PTCH mice chronically exposed to UV radiation^27^. In addition to animal experiments, clinical trials have extensively reported that ALA-PDT can cure the typical multiple AK lesions and prevent AKs and skin cancers^28^. Although a large number of studies have found that ALA-PDT can prevent photodamaged dermatosis, the exact mechanism remains unknown.

Based on these research results, we carried out experiments *in vivo* and *in vitro* to explore the underlying mechanisms. We pretreated the hairless mice skin and HaCaT cells with ALA-PDT, and then exposed them to UVB to detect DNA damage repair response. The results showed that ALA-PDT significantly reduced UVB-induced DNA damage by enhancing the repairing ability of DNA-damaged skin cells, suggesting ALA-PDT can inhibit UVB-induced photodamage.

## Material and Methods

### *In vivo* study

#### Animals

All animal experiments were approved by the Animal Use Committee of Nanjing Medical University and performed in accordance with the committee guidelines of Nanjing Medical University. In this study, we used 30 female BALB/c athymic nude mice, aged 4-6 weeks and weighing 18-22 g, purchased from Model Animal Research Center of Nanjing University. After acclimatization, the mice were randomly divided to 3 groups (10 animals in each group): Control group (no treatment), ALA-PDT-UVB group (10% ALA+12 J/cm^2^ red light+180 mJ/cm^2^ UVB), UVB group (saline+180 mJ/cm^2^ UVB).

#### ALA-PDT treatment and UVB irradiation

ALA was applied to a 4.0-cm^2^-area of dorsal skin using a piece of medical cotton soaked with ALA solution (10%; Fudan-Zhangjiang Bio-Pharmaceutical Co, China). UVB group used physiological saline as negative control. Subsequently, the treated areas were covered with layers of plastic wraps and black plastic sheets, secured with medical tape. Two hours later, these dressings were removed. The exposed tissues were irradiated by a PDT laser (XD-635AB; Fudan-Zhangjiang Bio-Pharmaceutical Co, China) with a wavelength of 635 nm, a duration of 10 min, and an energy density of 12 J/cm^2^. After treated with ALA-PDT or physiological saline and a 3-day interval, mice were irradiated with UVB, with a dose of 180 mJ/cm^2^. UVB irradiation was delivered by a UV fluorescent lamp (JCB35-24-01, Sigma, Shanghai, China). Irradiation output was monitored using a UV-meter (Philips, The Netherlands). During irradiation, mice were anesthetized with 0.4% chloral hydrate (0.1 ml/10g). The dorsal skin was collected 6 h, 24 h or 48 h after UVB irradiation for analysis.

#### Histology

Skin samples were sliced and fixed in 4% paraformaldehyde for routine paraffin embedding. For each specimen, a cascade of 6-μm sections were prepared, mounted on glass slides, and dried overnight at 37˚C to promote adhesion. After the sliced sections were treated with haematoxylin and eosin (H&E), the number of sunburn cells was observed.

#### TUNEL assay

TUNEL assay was performed in tissue sections using the One Step TUNEL Apoptosis Assay Kit (Beyotime Biotechnology, China) according to the manufacturer's instructions. Briefly, tissue sections were deparaffinized, rehydrated followed by antigen retrieval with digestion proteinase K (20 μg/ml) for 20 min at 37℃. Then slides were incubated with 5 μL TdT enzyme and 45μL fluorescein-dUTP for 60 min at 37℃. After washing, slides were double stained with 4′,6-diamidino-2-phenylindole (DAPI) to visualize the nuclei. For each group, the number of apoptotic cells and the sum of cells of five random fields were photographed and counted under the fluorescence microscope. The apoptotic index of the cells was calculated using the following formula: Apoptotic index = apoptotic cells/total cells × 100%.

#### Immunofluorescence (IF) analysis on CPDs

All specimens were fixed in 4% paraformaldehyde and then embedded in paraffin. After blocking endogenous peroxides and proteins, sections were incubated overnight at 4℃ with rabbit monoclonal primary antibodies against CPDs (1:500; Sigma, USA). Having been washed with PBS, the sections were incubated with appropriate FITC-labeled secondary antibodies (1:2000; Beyotime Biotechnology, China) at 37℃ for 1 h. Then the slides were observed and pictures were captured under a fluorescence microscope. All samples were examined by two investigators in a blind manner. Five randomly selected fields were counted, and the data represented the results in each group.

#### Immunohistochemical (IHC) analysis on Ki67

To evaluate epidermal proliferation, sections were stained with the proliferative marker Ki67. Briefly, sections were blocked in 10% goat normal serum for 20 min, incubated with rabbit monoclonal antibodies (1:1000; Abcam, Britain) overnight at 4℃. Sections were then washed and incubated with goat anti-rabbit secondary antibodies (1:2000; Beyotime Biotechnology, China) at 37℃ for 1 h. Subsequently, the 3-amino-9-ethyl carbazole (AEC) detection system (Bosterbio, USA) was used to visualize the staining. Sections of each group were photographed and counted under the microscope. The proliferation index was calculated using the following formula: Proliferation index = Ki67 positive cells/total cells × 100%.

### *In vitro* study

#### Cell culture

The immortalized human epidermal keratinocyte-like cell line (HaCaT) cells were cultured in Dulbecco's modified Eagle's medium (DMEM) supplemented with 10% fetal bovine serum (FBS) and 1% penicillin-streptomycin in a condition of 5% CO_2_ at 37℃. HaCaT cells were subcultured by trypsinization and used for subsequent processing.

#### Optimizing ALA concentration and red light laser irradiation dose

HaCaT cells were seeded into 96-well plates (1×10^5^ cells/ml). Cells treated with different ALA concentration (0.1, 0.25, 0.5, 0.75, 1, 1.5 and 2 mmol/L, respectively) were incubated in the darkness at 37^◦^C for 6 h, exposed to different doses of irradiation (0.5 J/cm^2^, 1.5 J/cm^2^, 3 J/cm^2^, 4.5 J/cm^2^, 6 J/cm^2^, 10 J/cm^2^, respectively) of red light laser (635 nm wave length, 50 mW/cm^2^) emitted from a semiconductor laser (XD-635AB, Shanghai Fudan-Zhangjiang Bio-Pharmaceutical Co., China). CCK-8 Kit (Beyotime Biotechnology, China) was used according to the manufacturer's instructions for cell viability assay.

#### UVB irradiation

HaCaT cells were cultured under a standard condition of > 70% confluency, and then irradiated by a UV fluorescent lamp (JCB35-24-01, Sigma, Shanghai, China) with a UVB dose of 60 mJ/cm^2^ 2 days after ALA-PDT or placebo pretreatment. All cells were treated with identical procedures. During the irradiation, the sham plate was shielded and the medium was removed and then covered with a thin layer of phosphate buffered saline (PBS). The intensity of UVB irradiation was measured using a UV-radiometer (Philips, The Netherlands).

#### Cell Viability measurement

Cell viability was measured with a CCK-8 kit (Beyotime Biotechnology, China). After the indicated treatment by ALA-PDT or UV irradiation, cells were washed for three times with PBS. Subsequently, 10 μL of CCK-8 was added to each well. After incubation for 1 h, cell density was determined through measuring the absorbance at 450 nm with a microplate reader (Termo Scientifc, USA).

#### Flow cytometry analysis on apoptosis

HaCaT cells that had been treated as above were harvested and washed with PBS, and double-stained by an Annexin V-FITC apoptosis detection kit (Beyotime Biotechnology, China) according to the manufacturer's instructions. Samples were incubated at room temperature for 15 min in the darkness with 50 μg/ml Annexin V-FITC and 10 μg/ml PI, and quantitatively analyzed by a FACScan flow cytometer (BD, Franklin Lakes, NJ, USA).

#### Optical microscopic evaluation on cell morphology

Cells were seeded into 6-well plates (1 × 10^6^ cells/well). After the last treatment, the medium was removed and washed for three times with PBS. DMEM containing 10% FBS was added and the cells were cultured for 24 h. The morphological changes were observed and photographed by an optical microscopy (U-ND25-2, Olympus, Japan).

#### Western blotting for p53^total^ and p53^Ser-15^ proteins detection

After the above mentioned treatments, HaCaT cells were subjected to Western blotting analysis. Cell proteins were extracted and quantified by a BCA kit, followed by electrophoretic separation on SDS-PAGE. After being transferred to PVDF membranes, samples were allowed to react with mouse anti-p53 antibodies (1:500; Beyotime Biotechnology, China) and rabbit anti-phospho-p53 (Ser15) antibodies (1:500; Beyotime Biotechnology, China). Then the membranes were incubated with HRP-conjugated anti-rabbit or anti-mouse IgG (1:4000; Beyotime Biotechnology, China) at room temperature for 2 h. Protein expression levels of p53^total^ and p53^Ser-15^ were visualized using an enhanced chemiluminescence detection system, and the data of optical density were analyzed using Image-J software. β-actin was used as an internal control.

#### Immunofluorescence analysis on CPDs

CPDs were measured with immunofluorescence 6, 24, 48 h after the pretreatment and UVB irradiation. HaCaT cells cultured on the coverslips were washed twice with isotonic PBS and fixed in ice-cold 4% paraformaldehyde solution in PBS for 30 min. The coverslips were washed for three times with PBS, and cells were permeabilized in PBS containing 0.5% Triton X-100 for 10 min. Then the cells were treated with 2 mmol/L HCl for 30 min to denature cellular DNA. After blocking nonspecific antibody-binding with 10% goat normal serum for 20 min, cells were incubated with rabbit anti-CPD antibodies (1:500; Sigma, USA) overnight at 4℃. The next day the cells were washed for three times with PBS. Then the cells were incubated with FITC-conjugated goat anti-rabbit IgG (1:1000; Beyotime Biotechnology, China) for 1 h at 37℃, and then washed for three times with PBS. The nuclei were stained with DAPI (Sigma, USA) for 5 min. Finally, the samples were examined under a fluorescence microscope and analyzed by comparing the percentage of CPDs positive cells.

### Statistics

Statistical analysis was performed by using Social Sciences (SPSS) software version 19.0. The statistical analyses were carried out by *t*-tests or One-way analysis of variance (ANOVA). *P* < 0.05 was considered to indicate statistical significance. Data were expressed as mean ± standard deviation (SD).

## Results

### *In vivo* study

#### ALA-PDT accelerated the removal of CPDs in hairless mice skin

**Figure 1:**
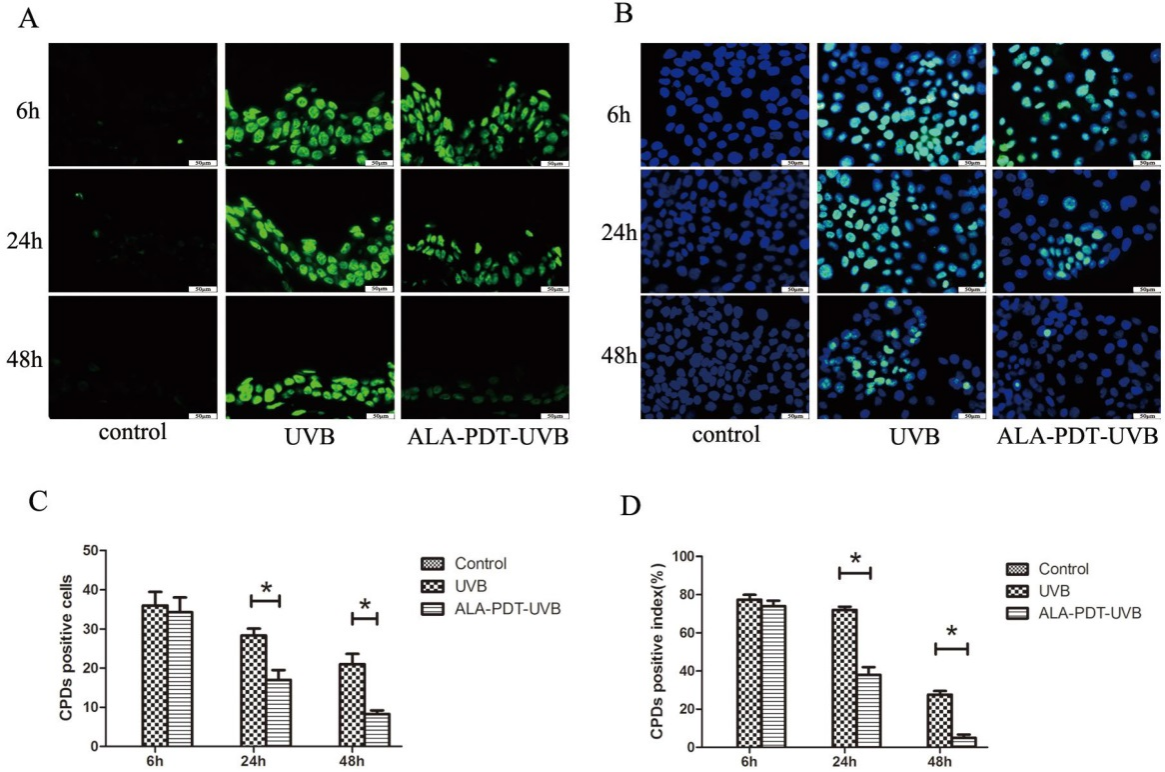
ALA-PDT accelerated the removal of CPDs in the dorsal skin of hairless mice and HaCaT cells after UVB irradiation. (A) CPDs positive cells in the epidermis of each group, stained by immunofluorescence staining and detected by fluorescence microscopy with × 400 magnifications. (B) CPDs positive cells in HaCaT cells of each group, stained by immunofluorescence staining and detected by fluorescence microscopy with × 400 magnifications. (C) The number of CPDs positive cells in the epidermis of each group. The number of CPDs positive cells in ALA-PDT-UVB groups was significantly decreased compared with UVB group at 6, 24 and 48 h after UVB irradiation (*P* < 0.05). (D) The number of CPDs positive cells in HaCaT cells of each group. The number of CPDs positive cells in ALA-PDT-UVB groups was significantly decreased compared with UVB group at 6, 24 and 48 h after UVB irradiation (*P* < 0.05). The symbol (*) indicates a significant difference (*P* < 0.05). Bar= 50μm, N= 3.

We observed no CPDs-positive nuclei in unirradiated sections and the largest number of CPDs, comparable to ALA-PDT-UVB and saline-UVB group, at 6 h after UVB irradiation (Figure 1A). In sections pretreated with ALA-PDT (or saline) and UVB irradiation, CPDs decreased with time. However, the removal rate of CPDs was obviously higher in ALA-PDT-treated sections. At 24 h, the number of initial CPDs-positive nuclei was 28.33±3.06 in saline-treated epidermis, compared with 17±4.36 in ALA-PDT-treated epidermis (*P* < 0.05) (Figure 1C). At 48 h, the number of CPDs-positive nuclei was 21±4.58 in saline-treated group, compared with 8.33±1.53 in ALA-PDT-treated group (*P* < 0.05) (Figure 1C).

#### ALA-PDT suppressed UVB-induced apoptosis of hairless mice skin cells

**Figure 2:**
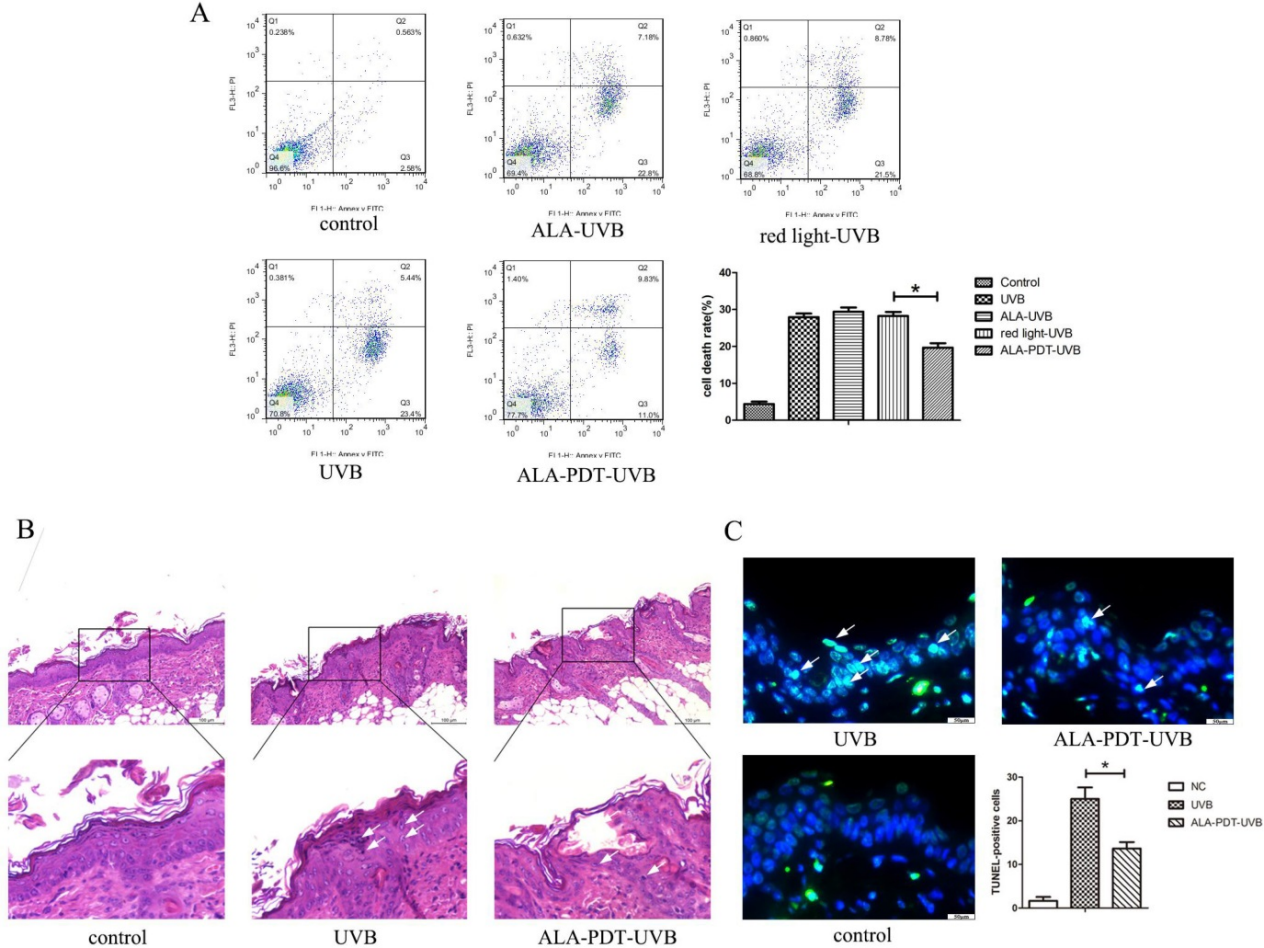
ALA-PDT suppressed UVB-induced apoptosis. (A) Evaluation of apoptosis of HaCaT cells by using annexin V/PI staining and flow cytometry. Annexin-V(-)/PI(-) cells are live cells, annexin-V(+)/PI(-) cells are early apoptotic cells and annexin-V(+)/PI(+) cells are late apoptotic or necrotic cells. The rate of apoptosis in ALA-PDT-UVB group was lower than that in UVB group (*P* < 0.05).(B) Sunburn cells in the epidermis of each group, stained by hematoxylin and eosin (H&E) staining and detected by light microscopy with × 200 magnifications. The number of sunburn cells in the epidermis of ALA-PDT-UVB group at 24 h after UVB irradiation was decreased compared with UVB group. (C) Apoptotic cells in the epidermis of each group, stained by TUNEL staining and detected by fluorescence microscope with × 400 magnifications. Compared with UVB group, the number of apoptotic cells in ALA-PDT-UVB group was significantly decreased (*P* < 0.05). The symbol (*) indicates a significant difference (*P* < 0.05). Bar= 100μm, N= 3.

Sunburn cells, a type of apoptotic keratinocytes characterized with pyknotic nuclei, condensed cytoplasm and intercellular edema, have a close correlation with UV radiation and can be used as an indicator for UV radiation-related damage. Histological evaluation of UVB-irradiated groups showed a large number of sunburn cells in the epidermis at 24 h after UVB irradiation (Figure 2B). The number of sunburn cells in the epidermis of ALA-PDT-treated group at 24 h after UVB irradiation were decreased compared with those in the UVB group (Figure 2B). The apoptotic cells were stained green by TUNEL staining. The non-irradiated group showed no TUNEL staining. UVB-irradiated groups showed TUNEL-positive apoptotic cells. Compared with UVB group, the proportion of apoptotic cells in ALA-PDT-treated group was significantly decreased (*P* < 0.05) (Figure 2C).

#### ALA-PDT inhibited UVB-induced hyperproliferation of hairless mice skin cells

**Figure 3:**
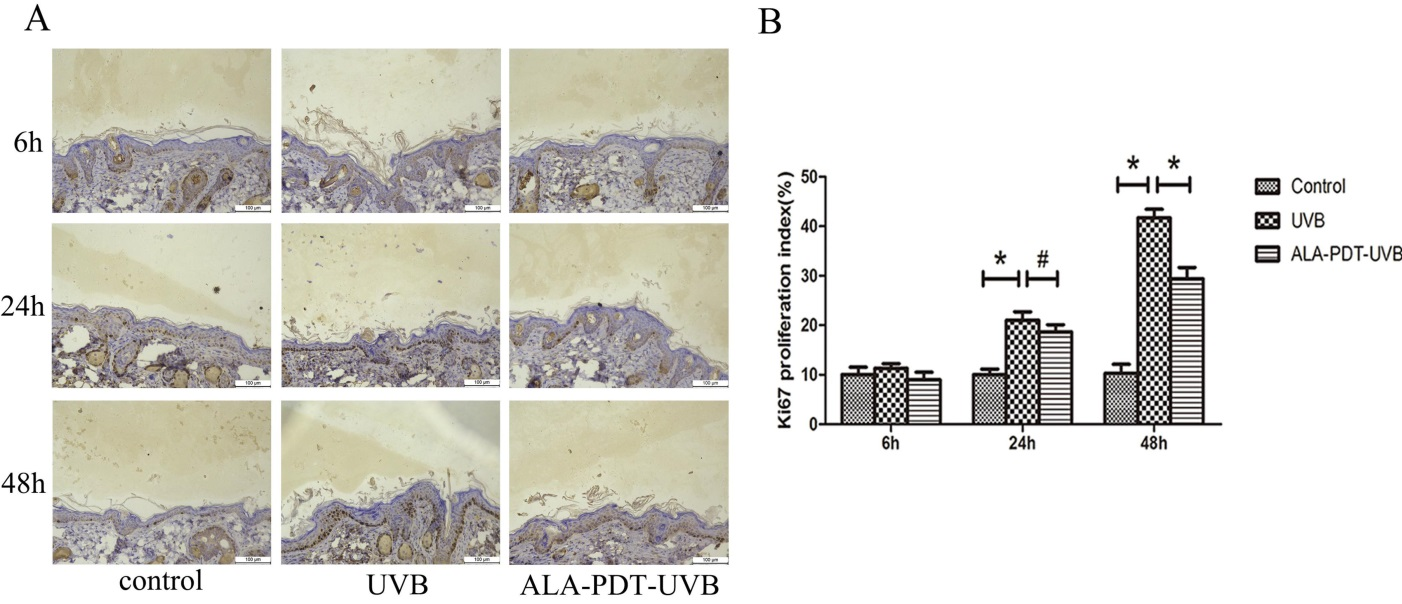
ALA-PDT pretreatment inhibited UVB-induced hyperproliferation in hairless mice skin. (A) Ki67 protein expression in the epidermis of each group at 6, 24, and 48 h after UVB irradiation, stained by immunohistochemical staining and detected by light microscopy with × 200 magnifications. (B) When compared with the UVB group, the number of Ki67 positive cells decreased in ALA-PDT-UVB group at 48 h after UVB irradiation (*P* < 0.05). The symbol (*) indicates a significant difference (*P* < 0.05). Bar= 100μm, N= 3.

To determine whether ALA-PDT can inhibit UVB-induced hyperproliferation, we evaluated the number of Ki67 positive nuclei in saline- or ALA-PDT-treated and then UVB-irradiated hairless mice skin. In unirradiated skin, the number of Ki67 positive nuclei was low (Figure 3A). No significant difference in the number of Ki67 positive nuclei was detected in saline- vs. ALA-PDT-treated skin at 6 h and 24 h after UVB irradiation. However, by 48 h in ALA-PDT-UVB group, the number of Ki67 positive nuclei was significantly less than in UVB group (*P* < 0.05) (Figure 3B).

### *In vitro* study

#### ALA-PDT protected HaCaT cells against UV-induced cell viability impairment

**Figure 4:**
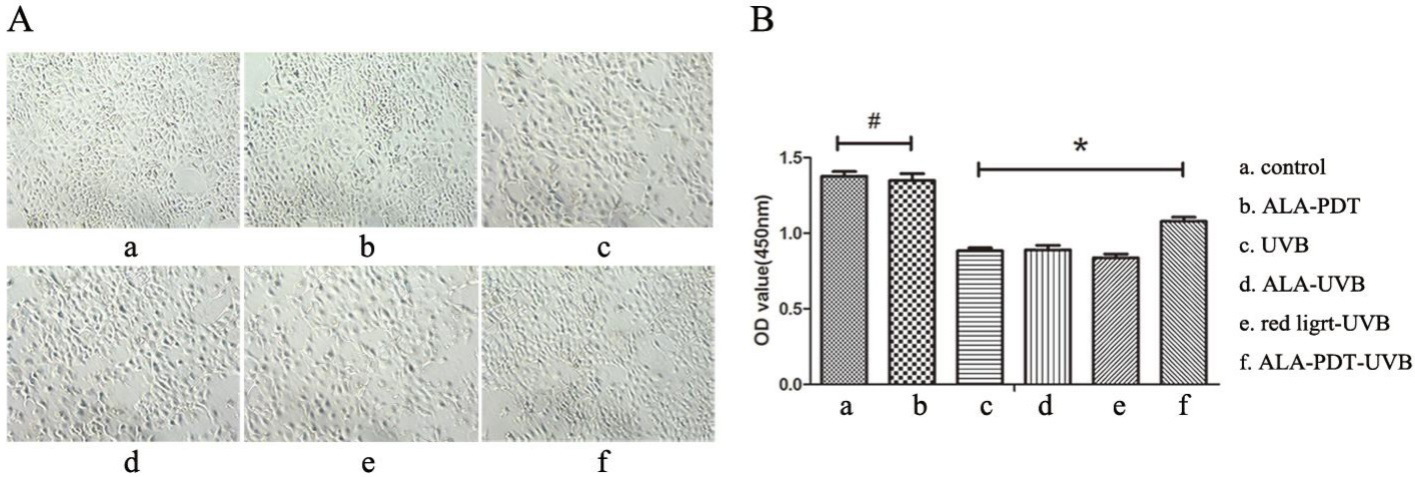
ALA-PDT protected HaCaT cells from UV-induced cell viability impairment. (A) Inverted phase contrast microscope observation of HaCaT cells. Cells of control group had the same shapes, clear boundaries, close arrangement. UVB group showed decreased cell number, decreased cell adhesion, increased intercellular space, rounded or irregular shapes, cell debris, and a large number of suspended cells. However, the cell morphology in ALA-PDT-UVB group was significantly normalized compared with UVB group. (B) Cell viability was analyzed by CCK-8. ALA (0.5 mmol/L) and low level of red light (3 J/cm^2^) had no effect on cell viability (*P* > 0.05). Cell viability of ALA-PDT-UVB group was higher than that of UVB group (*P* < 0.05). The symbol (*) indicates a significant difference (*P* < 0.05).

To observe whether ALA-PDT leads to the apoptosis of HaCaT cells, we performed cck-8 assay to quantify the cell viability. Results showed that 0.5 mmol/L of ALA and 3 J/cm^2^ of red light did not affect the vitality of cells, and compared with the control group, the difference was not significant (*P* > 0.05) ( Figure 4B). HaCaT cells viability of UVB group was significantly lower than that of ALA-PDT-treated group (*P* < 0.05), indicating that ALA-PDT significantly decreased UVB-induced cytotoxicity (Figure 4B).

#### ALA-PDT accelerated the removal of CPDs in HaCaT cells

After UVB irradiation, the CPDs-positive cells were stained with immunofluorescence staining. No CPDs-positive cells were found out of HaCaT cells in the control group. UVB-irradiated groups showed a large number of CPDs-positive cells, with the peak at 6 h after UVB irradiation. Then, at 24 h and 48 h after UVB irradiation, the proportions of CPDs-positive cells in ALA-PDT pretreatment group were significantly decreased compared with UVB group (*P* < 0.05)( Figure 1B, D).

#### ALA-PDT increased the levels of p53^total^ and p53^Ser-15^ proteins in HaCaT cells

**Figure 5:**
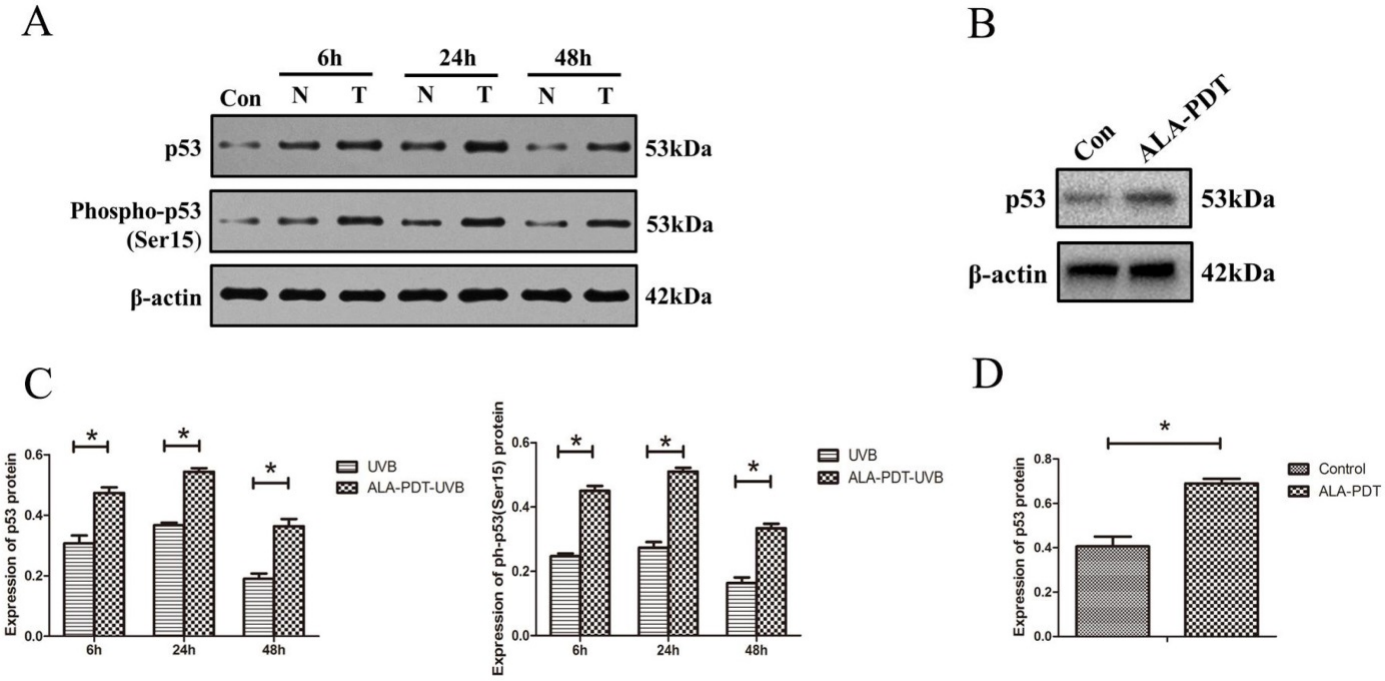
ALA-PDT up-regulated and activated p53 in HaCaT cells. (A) Western blotting for detection of p53^total^ and p53^ser-15^ protein in HaCaT cells of each group at 6, 24, and 48 h. Con: control group, N: UVB group, T: ALA-PDT-UVB group. (B) Expression of p53^total^ in HaCaT cells at 24 h after ALA-PDT treatment (0.5 mmol/L + 3 J/cm^2^). (C) (D) Protein expressions were quantified by β-actin as a control. The ratio of gray scale values represents the ratio of the amount of protein to interest/actin. The symbol (*) indicates a significant difference (*P* < 0.05). N= 3.

To determine whether ALA-PDT can increase the levels of p53 protein level and serine-15 phosphorylation *in vitro*, we processed HaCaT cells for both p53^total^ and p53^Ser-15^ Western blotting detection. The results showed that UVB-irradiated groups had significantly higher level of p53^total^ and p53^Ser-15^ expression compared with unirradiated group (*P* < 0.05) (Figure 5A). Further, as shown in Figure 5B, C, D, the expression of p53^total^ and p53^Ser-15^ in the ALA-PDT-treated samples were significantly higher than those in samples untreated with ALA-PDT (*P* < 0.05).

#### Inverted phase contrast microscope observation

Inverted microscope showed cells of control group had the same shapes, clear boundaries and close arrangement. UVB group showed a decreased cell number decreased cell adhesion, increased intercellular space, rounded or irregular shapes, debris, and a large number of suspended cells. However, the cell morphology of ALA-PDT group was significantly normalized compared with UVB group (Figure 4A).

#### ALA-PDT suppressed UVB-induced apoptosis in HaCaT cells

Annexin-V (-) / PI (-) cells are live cells, Annexin-V (+) / PI (-) cells are early-stage apoptotic cells and annexin-V (+) / PI (+) cells are late-stage apoptotic or necrotic cells. Apoptosis rate was expressed as a percentage of apoptotic cells in total cells. As shown in Figure 2A, the percentage of apoptotic cells in ALA-PDT pretreatment group was 27.95±1.71, significantly lower than 19.69±2.09 in the UVB group (*P* < 0.05).

## Discussion

Due to its ability of enhancing dermal remodeling and resolves chronic inflammation, ALA-PDT has been used to treat skin photodamages, including actinic keratoses, squamous cell carcinomas, basal cell carcinomas and photoaging^17^, ^20^, ^29^-^31^. Inge Seubring *et al.* treated AK patients with “lesion-by-lesion” methyl aminolevulinate photodynamic therapy (MAL-PDT) and field MAL-PDT. Nine months later, field MAL-PDT significantly reduced AK lesions compared with lesion-by-lesion MAL-PDT^32^. ALA and its methyl esters (methyl 5-aminolevulinate) are the most effective topical photosensitizers approved for treating actinic keratoses^21^, ^22^. ALA esters can notably improve skin uptake^17^, ^33^, ^34^, but the porphyrin fluorescence of ALA and ALA methyl esters showed the same microscopical spatial distribution^35^. As to the ability of reducing AK, ALA and ALA esters showed no difference. Although a large number of recent studies have proven that ALA-PDT can prevent skin tumors, the underlying mechanisms are unknown. To explore these mechanisms, we carried out *in vivo* animal and* in vitro* cell experiments.

ALA-PDT, functioning as photosensitizers, interacts with light and oxygen, producing reactive oxygen species (ROS) that plays a leading role in the mechanism of ALA-PDT^36^, ^37^. Low-level ROS can act as cellular signaling messengers by modifying protein structures and functions, activating dermal fibroblasts and increasing cellular proliferation, just as in treating skin rejuvenation^38^. Through disrupting cellular processes by non-specifically attacking proteins, lipids and DNA, high-level ROS can decrease cellular proliferation and increase cytotoxicity, just as in treating skin cancers^39^. In our study, low concentration of ALA (0.5 mmol/L) and low level of red light (3 J/cm^2^) had no effect on cell viability, though ROS production was induced.

UVB irradiation directly damages the skin's cellular DNA, through which CPDs and 6-4pps are generated. Outnumbering 6-4pps and more difficult to be repaired, CPDs are always regarded as the principal product of photodamage^10^, ^40^. CPDs, if not removed by the nucleotide excision repair pathway in time, may lead to DNA mutation^41^. Therefore, detecting the generation and removal of UVB-induced CPDs is useful for assessing the severity of DNA damage and repair. In this study, we found that UVB-induced CPDs in ALA-PDT group were removed more rapidly than UVB group. Some antioxidants, such as polyphenols and oligopeptides, can promote the removal of CPDs^42^, ^43^ mainly through absorbing UVB and clearing oxygen free radicals, and reduce the damage caused by oxygen free radicals in CPDs nucleotide excision repair pathway.^42^ Barbara A. Gilchrest *et al.* previously demonstrated T-oligos accelerated the clearance of CPDs, through a protective DNA damage response induced by activation of P53^44^.

Inducible DNA repair, termed as SOS response, was first found in bacteria. In essence, following acute sublethal DNA damage is a transient increase of DNA repair rate^45^. Barbara A. Gilchrest *et al.* demonstrated the existence of inducible DNA damage response that increased DNA repair capacity and reduced the impact of environmental carcinogens^44^, ^46^, ^47^. They reported that in vitro thymidine dinucleotide activated p53 and increased the ability of skin cells (endogenous DNA, to be specific) to repair subsequent UV-induced DNA damage^48^.

Further investigation on the expression of p53 after UVB radiation was conducted to substantiate the inducible DNA damage response of ALA-PDT after UVB radiation. As a transcription factor, p53 protein regulates the expressions of a series of downstream target genes to maintain genomic integrity through transient cell cycle arrest for DNA repair, and if the DNA damage could not be repaired correctly, p53 would induce programmed cell death or apoptosis^15^. In our study, after HaCaT cells were pretreated with ALA-PDT and then irradiated with UVB, ALA-PDT induced and activated p53 within 24 h and significantly increased p53 after UVB exposure. This further indicates that ALA-PDT may induce a protective DNA damage response through activating P53.

To verify whether ALA-PDT improved the skin cells' ability of repairing damaged DNA, we observed the epidermal DNA damage through strand breaks (TUNEL assay) and sunburn cells (H&E). Exposure to short-term and high-dose ultraviolet radiation can damage the DNA of skin cells and induce the apoptosis of keratinocytes, morphologically turning the later into sunburn cells. Exposure duration, UV dose and light work together to decide the development of sunburn cells. After UVB (200 mJ/cm^2^), it needs at least 24 h for sunburn cell to come into being^49^. Our previous study also found that the formation of sunburn cells in human epidermis reached the highest level 24 h after UVB (180 mJ/cm^2^) irradiation^42^. DNA strands break prior to the formation of morphologically distinct sunburn cells^50^. These explained why only a few sunburn cells were observed by H&E staining 24 h after UVB, but TUNEL detected a large number of apoptotic cells. More cell experiments showed that ALA-PDT increased cell viability and decreased apoptosis after UVB irradiation. These results indicate that ALA-PDT reduces the generation of apoptotic cells.

UV irradiation results in a dose-dependent arrest of epidermal proliferation and then hyperplasia, which exerts a protective effect by prolonging the path of photons to putative stem cells in the basal layer of the epidermis during UV exposure^41^, ^51^, ^52^. However, if DNA replication and cell division occur when DNA repair is still on, the risk of mutation rises.^10^ Simin Arad *et al.* reported that T-oligos-induced protective DNA damage response could inhibit UV-induced epidermal proliferation^44^. Consistent with this, we also found that ALA-PDT significantly inhibited the epidermal proliferation.

In conclusion, our study demonstrates that ALA-PDT can induce a protective DNA damage response through activating p53 and enhancing DNA repair capacity. This enhanced repair capacity can inhibit epidermal proliferation, reduce the generation of apoptotic cells and accelerate the removal of CPDs, which work together to protect skin cells against UVB-induced photodamage. However, whether ALA-PDT can prevent sunlight-induced tumors in vitro experiments has not been confirmed. In addition, the specific method of using ALA-PDT to prevent sunlight-induced diseases remains to be created.

## Abbreviations

UV: Ultraviolet
ALA-PDT: 5-aminolaevulinic acid-based photodynamic therapy
AK: actinic keratosis
CPDs: cyclobutane pyrimidine dimers
CCK: Cell Counting Kit
ROS: reactive oxygen species
FBS: fetal bovine serum
PBS: phosphate buffered saline
OD: Optical density
SD: standard deviation
H&E: haematoxylin and eosin
DAPI: 4',6-diamidino-2-phenylindole
DMEM: Dulbecco's modified Eagle's medium
